# Expert Opinion on the Diagnosis and Management of Male Hypogonadism in India

**DOI:** 10.1155/2023/4408697

**Published:** 2023-02-22

**Authors:** Sanjay Kalra, Jubbin Jacob, A. G. Unnikrishnan, Ganapathi Bantwal, Abhay Sahoo, Rakesh Sahay, Sushil Jindal, Madhu Sudan Agrawal, Nitin Kapoor, Banshi Saboo, Mangesh Tiwaskar, Kapil Kochhar

**Affiliations:** ^1^Department of Endocrinology, Bharti Hospital, Karnal 132001, Haryana, India; ^2^Department of Endocrinology, Christian Medical College and Hospital, Ludhiana 141008, Punjab, India; ^3^Chellaram Diabetes Institute, Pune 411021, Maharashtra, India; ^4^Department of Endocrinology, St Johns Medical College, Bengaluru 560034, Karnataka, India; ^5^Department of Endocrinology, Institute of Medical Sciences and SUM Hospital, Bhubaneswar 751003, Odisha, India; ^6^Department of Endocrinology, Osmania Medical College, Hyderabad 500095, Telangana, India; ^7^People's Medical College and Research Centre, Bhopal 462037, Madhya Pradesh, India; ^8^Department of Urology, Global Rainbow Hospita, l, Agra 282007, Uttar Pradesh, India; ^9^Department of Endocrinology, Diabetes and Metabolism, Christian Medical College, Ida Scudder Road, Vellore 632004, Tamil Nadu, India; ^10^Baker Heart and Diabetes Institute, Melbourne, Australia; ^11^Department of Medicine, Dia Care, Ahmedabad 380015, Gujarat, India; ^12^Department of Medicine, Shilpa Medical Research Centre, Mumbai 400068, Maharashtra, India; ^13^Andrology Clinic, Indore, Madhya Pradesh, India

## Abstract

Male hypogonadism (MH) is a clinical and biochemical syndrome caused by inadequate synthesis of testosterone. Untreated MH can result in long-term effects, including metabolic, musculoskeletal, mood-related, and reproductive dysfunction. Among Indian men above 40 years of age, the prevalence of MH is 20%–29%. Among men with type 2 diabetes mellitus, 20.7% are found to have hypogonadism. However, due to suboptimal patient-physician communication, MH remains heavily underdiagnosed. For patients with confirmed hypogonadism (either primary or secondary testicular failure), testosterone replacement therapy (TRT) is recommended. Although various formulations exist, optimal TRT remains a considerable challenge as patients often need individually tailored therapeutic strategies. Other challenges include the absence of standardized guidelines on MH for the Indian population, inadequate physician education on MH diagnosis and referral to endocrinologists, and a lack of patient awareness of the long-term effects of MH in relation to comorbidities. Five nationwide advisory board meetings were convened to garner expert opinions on diagnosis, investigations, and available treatment options for MH, as well as the need for a person-centered approach. Experts' opinions have been formulated into a consensus document with the aim of improving the screening, diagnosis, and therapy of men living with hypogonadism.

## 1. Introduction

Male hypogonadism (MH) is a clinical and biochemical syndrome characterized by testosterone deficiency with associated signs and symptoms. It is caused by the failure of the testis to synthesize physiological levels of testosterone and a normal number of spermatozoa due to the disruption of one or more levels of the hypothalamic-pituitary-testicular axis. MH can occur due to an intrinsic defect of the testes (primary hypogonadism), in which the Leydig, Sertoli, and germ cells are impaired, or at the level of the hypothalamus or pituitary gland (secondary hypogonadism), in which the testes lack gonadotropic stimulation by luteinizing hormone (LH) and follicle-stimulating hormone (FSH) [1].

The global prevalence of MH varies between 6% and 12% based on the study population and level of diagnostic rigor [2, 3]. In India, the prevalence ranges from 20% to 29% in adult men of average age 40–62 years [4, 5]. Untreated MH can lead to long-term effects, such as sexual dysfunction, anemia, osteoporosis and fracture, myopathy and frailty, tender gynecomastia, psychosocial impairments, and reduced quality of life. There appears to be an association of MH with obesity, metabolic syndrome, and type 2 diabetes mellitus (T2DM) [6], although the causal relation is yet to be elucidated [7].

Although multiple guidelines are available from various international societies, such as the Endocrine Society (USA), the Society for Endocrinology Guidelines (UK), and the Endocrine Society of Australia, there are no Indian guidelines available for the management of MH; therefore, the Endocrine Society guidelines are most frequently used by clinicians across India [2, 8, 9].

In order to garner expert opinion on several aspects of hypogonadism, including screening and diagnosis (based on medical history and biochemistry, including serum testosterone levels) and management, including the need for a person-centered approach, five virtual advisory board meetings, one at the national level and four at the regional level, involving endocrinologists, diabetologists, urologists, and andrologists from across India, were convened. A literature review was performed based on data from the PubMed database using the keywords “India,” “burden,” “male hypogonadism,” “testosterone,” “andropause,” “aging male,” “late-onset,” “testosterone replacement therapy,” “guidelines,” and “management.” During the advisory board meeting, in addition to the interactive discussion, a qualitative question-and-answer-based format was used to facilitate the discussion. Key expert opinions were formulated based on group discussions, and a consensus document was developed.

## 2. Diagnosis of Male Hypogonadism

### 2.1. Public Attributes

In India, men aged >60 years old and those who experience sexual dysfunction, loss of energy, loss of libido, loss of interest in life, and loss of muscle mass typically do not seek medical counsel as they feel that these conditions are age-related problems and are not associated with a deficiency in testosterone levels that can be treated (Table 1). Androgen deficiency increases with age in healthy men. The diurnal variation in total testosterone and free testosterone over the aging process is not significantly different and is explained by the age-related increase in circulating levels of sex hormone-binding globulin (SHBG), which reduces the proportion of free testosterone. In healthy men, the age-related decline in testosterone is not accompanied by an increase in LH, which implies that the decline is mainly due to primary testicular failure compensated for by increased LH secretion [10].

The incidence of hypogonadism in middle-aged men is 2.1%–12.8%, with late-onset hypogonadism (LOH) occurring in 2%–15%. Among Indian men above 40 years of age, the prevalence is 20%–48% [4, 5]. In a study of men with T2DM, 20.7% were found to have hypogonadism [11]. Men with diabetes frequently complain of erectile dysfunction (ED) and loss of libido. Obese men present with low energy, tiredness, and lethargy [12]. The typical clinical presentation is a 40- to 50-year-old man who is centrally obese and complains of ED along with all other symptoms. The overlap between various symptoms, etiologies, risk factors, and confounding factors tends to reduce clinical suspicion of MH. In this regard, it has been observed that a higher proportion of men with moderate and severe LOH, as defined by the European Male Aging Study, had diabetes, metabolic syndrome, and cardiovascular disease compared with eugonadal men [13, 14].

### 2.2. Comorbidities Associated with Male Hypogonadism

Surgical comorbidities such as cryptorchidism, testicular trauma, and torsion, as well as radiotherapy to the gonads, may be associated with hypogonadism. Varicosity causes a decrease in sperm count; although it is not a cause, it may be associated with hypogonadism [10].

Medical comorbidities that may cause MH include obesity, malignancies, HIV/AIDS, chronic kidney disease, chronic heart failure, and chronic liver disease. Psychological correlates of hypogonadism include depression, obsessive-compulsive neurosis, and bipolar disorders [10], whereas endocrine causes include hypothyroidism, hypercarotenemia, autoimmune polyglandular syndrome type 2, Cushing syndrome, and hemochromatosis. If hormonal diseases such as Klinefelter syndrome, Kallmann syndrome, hypergonadotropic hypogonadism, or hyperprolactinemia are suspected to be the cause and the clinical features are suggestive of secondary causes, a thorough workup might be required.

Some features of hypogonadism, such as lethargy, tiredness, and fatigue, are nonspecific and must be probed further. Iatrogenic causes include the use of steroids and anabolic steroid abuse, especially in men who excessively undertake muscle-building exercises. Alcoholism is also a major contributing factor in hypogonadism [10].

### 2.3. Screening for Male Hypogonadism

Suspicion and initial screening of MH are based on the clinical tolls of history-taking and examination, along with the assessment of surrogate laboratory and imaging parameters. Biochemical assessment has a secondary role in the diagnosis of MH. This is in stark contradiction to the approach followed in diabetes and hypothyroidism, where target populations are screened even if asymptomatic [15, 16].

The screening and assessment of MH depend on the intensity with which clinicians investigate patients using questionnaires and the depth of the medical history assessment (Table 2). The International Index of Erectile Function (IIEF) 5 questionnaire [17] is a screening tool that helps in reducing the burden on laboratories and augmenting the findings of abnormal laboratory tests when a patient complains of ED or loss of libido [11]. Hormonal assessment is advised only in patients with moderate or moderate-to-severe symptoms.

### 2.4. Investigations for Male Hypogonadism

Usually, total testosterone levels are assessed to diagnose MH. When total testosterone levels are equivocal, free testosterone levels may also be assessed to confirm the diagnosis of MH. In conditions where SHBG levels are high, particularly in elderly subjects in whom SHBG levels increase with aging, free testosterone tests are advised [18]. A serum testosterone value confirms the diagnosis of MH and serves as a monitoring tool during therapy.

Testosterone release in adults usually exhibits circadian variation, with peak levels occurring in the early morning and the lowest values (up to 60% lower) being observed in the evening. Given the degree of diurnal variation in circulating testosterone levels, serum testosterone levels should be tested in morning samples drawn in the fasted state, but nonfasting levels are also acceptable [18].

Primary and secondary hypogonadism may have a functional deficiency similar to LOH. The levels of LH, FSH, and prolactin need to be checked to differentiate secondary (pituitary/hypothalamic) hypogonadism from primary hypogonadism. Primary hypogonadism is mainly due to testicular failure and may have a number of causes, such as Klinefelter's syndrome, undescended testicles, mumps orchitis, hemochromatosis, testicular trauma, or chemotherapy/radiation therapy [18].

A combination of sexual symptoms and serum testosterone levels <231 ng/dL defines LOH. Based on several international guidelines and recommendations, Kim and Moon provided an algorithm for the diagnosis of LOH, as shown in Table 3 [19].

There are no absolute testosterone levels below which a man can unambiguously be considered to have hypogonadism (Table 4). The Endocrine Society recommends 300 ng/dL, while the American Association of Clinical Endocrinology recommends 200 ng/dL as the lower limit of normal total testosterone. The International Society of Andrology, International Society for the Study of the Ageing Male, European Association of Urology (EAU), European Academy of Andrology, and American Society of Andrology recommendations suggest 230 ng/dL as the limit below which patients will usually benefit from TRT [20]. However, in men with a high prevalence of obesity or T2DM and those with conditions that affect SHBG levels, low total testosterone levels cannot reliably predict biochemical hypogonadism. Thus, total testosterone levels less than 150 ng/dL are considered to indicate severe hypogonadism, which in turn can predict low free testosterone. Conversely, total testosterone levels between 280 and 350 ng/dL are not sensitive enough to reliably exclude hypogonadism; the level of total testosterone must exceed 350–400 ng/dL to reliably predict normal free testosterone [21]. A borderline serum testosterone value may be repeated for confirmation or managed with a therapeutic trial after informed and shared decision-making.

Anemia in men with hypogonadism is partly attributed to declining testosterone levels in aging hypogonadal men and partly due to the effects of testosterone on erythropoietin and erythroid progenitor cells [22]. Testosterone-replacement therapy can help restore hemoglobin levels but also increases hematocrit, considering that a direct relation has been suggested between testosterone dosage and the incidence of erythrocytosis. While elevated hematocrit is beneficial in hypogonadal men with anemia, it can also increase blood viscosity and cause exacerbation of vascular disease, particularly in the elderly [22]. Thus, during treatment, the primary aim should be to avoid erythrocytosis of overreplacement and anemia of underreplacement, if necessary overriding ideal testosterone targets.

## 3. Management of Male Hypogonadism

### 3.1. Approach toward Management

The management of MH should be done in a person-centric manner. The treatment should be based on the clinical background, associated comorbidities, expected response, and side effect tolerance profile of a given individual [23]. For patients with confirmed hypogonadism (either primary or secondary testicular failure), TRT is recommended. Patients with classical hypogonadism are initiated on testosterone therapy, starting with 100 mg monthly injections for 3 months, followed by 250 mg every 3 weeks. Serum testosterone is tested at 1.5 weeks and at the end of 3 weeks. If total testosterone levels are still low at the end of 3 weeks, treatment may be changed to twice weekly [11, 19].

Oral testosterone capsules (40 mg, 3 times daily) are recommended in patients with LOH or functional hypogonadism based on findings from certain clinical studies [24]. However, there are no data to support the effectiveness of testosterone treatment in the management of functional central hypogonadism (or nongonadal illness), for which lifestyle measures are proven to be effective. In this regard, Australian guidelines do not recommend testosterone therapy, whereas the USA Endocrine Society guidelines recommend it at clinician discretion, and the UK guidelines recommend it for ED refractory to first-line treatments, anemia, or low bone density [2, 8, 9, 25].

Testosterone gel (1% at a dose of 5–10 g) is used in men who prefer this mode of therapy and want stable levels of testosterone and in children who present with micropenis. Long-acting injectables (1000 mg undecanoate) are economical and offer stable circulating levels of testosterone, but they are not preferred by some patients as they are painful [19]. Pain can be minimized if the ampoule is first prewarmed to body temperature and the injection is administered slowly over 1–2 minutes by experienced nurses. Postinjection pain requires only infrequent analgesic use and causes little disturbance in daily living activities [26].

The ISSM, British Society for Sexual Medicine (BSSM), and EAU recommend diagnosing MH and considering offering TRT on the basis of serum total testosterone <231 ng/dL, with samples taken in the morning on two or more separate occasions (spaced at least 4 weeks apart according to the BSSM and 1 week apart according to the ISSM). The American Urological Association suggests a higher cutoff of 300 ng/dL to initiate therapy, while the Canadian Medical Association Journal does not specify any predetermined serum total testosterone threshold levels, nor does it advise repeat sampling if levels are low on initial measurement [27].

Prostate cancer is an absolute contraindication for TRT. Any person above 40 years of age with a family history of prostate cancer should be recommended to undergo prostate-specific antigen (PSA) tests. Men with breast cancer should not be treated with testosterone preparations. Baseline hematocrit and sleep apnea can be precipitated by exogenous androgens. Packed cell volume also should be investigated in these patients. Hematocrit >50%, an untreated 3-year history of sleep apnea, erythrocytosis, and untreated heart failure are some of the other contraindications to TRT. Androgens can have sodium-retention properties. Severe lower urinary tract symptoms with high scores because of BPH or hyperplasia might also need to be taken into consideration. Erythrocytosis can be associated with testosterone ester preparations, and it is also directly related to venous thromboembolic disease. Patients with erythrocytosis should be monitored at baseline and regularly after 3–6 months after initiating testosterone therapy. Patients with the chronic obstructive pulmonary disease have raised hematocrit, and using testosterone therapy for MH in these patients is problematic [28].

According to the Endocrine Society, BSSM, European Male Ageing Study, and International Society of Urology, TRT is also not recommended in men with a palpable prostate nodule or induration, as well as in men with a PSA level > 4 ng/mL or a PSA > 3 ng/mL combined with a high risk of prostate cancer. A baseline PSA of <2 ng/mL is recommended before initiating therapy. Follow-ups should be scheduled at 3 months, 6 months, 12 months, and annually thereafter. A digital rectal examination can be performed annually, and transliteral ultrasound is always recommended to visualize any nodule or induration [28].

### 3.2. Testosterone Formulations

Optimal TRT remains a considerable challenge due to the need for individually tailored therapeutic strategies. Oral delivery is challenging due to rapid first-pass metabolism and a short half-life (Table 5). To date, testosterone derivatives have been developed to enhance intrinsic androgenic potency, prolong the duration of action, or improve the oral bioavailability of synthetic androgens [29]. Various formulations of testosterone are available and include intramuscular injections, oral and buccal preparations, and gels (Table 6).

For oral preparations, the current dosage is thrice a day [28]. Oral preparations have relatively less bioavailability and have to be taken with high-fat meals. Oral preparations are expensive and need multiple doses, and their efficacy might improve if higher doses are used. The thrice a day frequency of administration may make this medication unappealing to some patients. As the patient is required to take these medications for a prolonged period of time, and usually these patients have some comorbidities, such as hypertension and diabetes mellitus, reducing the pill count may improve medication compliance and adherence. Oral undecanoate preparations do not affect the liver as they are not absorbed via the portal system but by the lymphatic system, thereby bypassing the hepatic system [28].

Gels are associated with swift onset of action and maintenance of smooth levels, but they are expensive and may not be welcomed by some men who view them as messy. Testosterone esters are available with a duration of action of 21–28 days; they are economical but are associated with erratic testosterone levels. While there may be a supraphysiological level of testosterone about a week to 10 days after injection, there may also be a trough of suboptimal or below normal serum testosterone towards the tail end of the 3–4 week long period. Various injectable preparations are available. If the patient accepts injectable testosterone as a therapy, one may start with short-acting aqueous formulations given once a week to once a fortnight. These are associated with the rapid achievement of optimal serum testosterone and also help in the rapid resolution of symptoms. The patient may continue with this form or may choose to shift to longer-acting preparations. Testosterone undecanoate can be used as a 3-monthly injection. However, the volume of injectable testosterone is 4 mL, and it requires a trained medical professional to inject the preparation. All testosterone ester injections are associated with a relatively higher level of pain and discomfort. All these factors should be discussed with the patient at length who can then chose the testosterone preparation that suits him the most. In general, it is advisable to begin with gel, oral preparation, or aqueous injection while initiating therapy (Table 5). Once the patient has stabilized, it can be discussed whether he would like to continue the same or shift to a long-acting injection [23, 28].

Swerdloff and Duley evaluated the safety and efficacy of a novel formulation of oral TU in a long- and short-term phase 3 trial in men with MH [30]. Men aged 18–65 years with hypogonadism, defined by verified low morning serum total testosterone  <300 ng/dL and a history of signs and/or symptoms consistent with hypogonadism, were recruited into a 365-day (trial I) or 105-day (trial II), randomized, multicenter trial. Patients were naïve to androgen replacement therapy or had had an adequate washout of previous androgen replacement therapies. They were randomized at a ratio of 1 : 1 to receive oral TU (*n* = 161) or testosterone gel (*n* = 160) in trial I, and at a ratio of 3 : 1 to receive oral TU twice daily (Jatenzo® (*n* = 166)) or topical testosterone (Axiron® (*n* = 56)) in trial II. Oral TU significantly (*p* < 0.0001) improved all psychosexual daily questionnaire parameters in trials I and II. In trial I, lean mass increased by 3.2 (2.7) kg, fat mass decreased by 2.4 (3.6)  kg (both *p* < 0.0001), and bone density improved in the hip (0.012 (0.0225)  g/cm^2^) and spine (0.018 (0.0422)  g/cm^2^) after 365 days (both *p* < 0.0001). The improvement in fat mass especially becomes relevant in the south Asian phenotype, where we find a significantly higher proportion of body fat even in individuals with normal weight and especially in the visceral compartment [31, 32]. Oral TU was neither associated with liver toxicity nor did it cause an elevation in high-sensitivity C-reactive protein or lipoprotein-associated phospholipase *A*_2_ (cardiovascular safety biomarkers) after 365 days of therapy. Therefore, the new oral TU formulation was found to be safe and effective and to represent a significant therapeutic advantage for the treatment of hypogonadal men [30].

There is yet no consensus on the effect of higher doses of TRT on hypogonadism in Indian men. Data on the effect of such doses on the prostate, cardiac, and other health parameters are unavailable. Besides, the effectiveness of TRT on Indian subjects also needs to be evaluated.

## 4. Current Challenges and Way Forward

Challenges in the diagnosis of MH are the lack of specific symptoms; the role of obesity, metabolic syndrome, and diabetes mellitus; and low testosterone values. Further, there is no consensus on how to approach patients for diagnosis; the signals for suspicion of hypogonadism, time of sample collection, and therapy as guidelines vary in their recommendations for these factors.

Testosterone cutoff values also differ across different guidelines. While some guidelines suggest that values < 300 ng/dL should be considered low, it is not yet determined whether they are related to loss of libido or ED. Sometimes patients with testosterone values < 200 ng/dL and loss of libido have benefitted from treatment.

The biggest challenge for patients is the lack of awareness about the type of expert to be consulted for sexual dysfunction problems. Moreover, patients' ignorance about or lack of comfort in describing their symptoms may impact diagnosis. In India, late presentation of hypogonadism is common because patients are hesitant to discuss their illnesses. Moreover, a history of long-term diabetes, obesity, congestive heart failure, obstructive sleep apnea, or other comorbidities is not considered. On the other hand, hypogonadism may be a cause of anemia and reduced responsiveness to erythropoiesis-stimulating agents in men with chronic kidney disease [33]. Furthermore, undiagnosed clinical hypogonadism has been identified as a common cause of osteoporosis in men, which increases the risk of fracture [34].

In a survey among men presenting for urologic evaluation, half of the respondents were unsure of the risks of TRT. Although 43% of all respondents reported an interest in TRT, only half of them had a clinical diagnosis of hypogonadism. These findings indicate an ongoing need for patient education regarding TRT [35].

Physicians are accurate and reliable sources of credible information for patients. However, physicians might not be aware that disparities exist between patient knowledge and available educational resources. Recognizing the knowledge gap is an important first step in a series of changes that should occur at the physician and patient levels [36]. The need to educate physicians about the management of hypogonadism is also urgent. Because physicians routinely treat diabetes mellitus, thyroid disorders, and other comorbidities, they should also gain expertise in the diagnosis of MH and refer suspected patients to endocrinologists. Educating physicians about hypogonadism would prove beneficial in improving diagnostic accuracy, increasing endocrinologist referrals, and increasing the number of patients receiving therapy, thus improving patient outcomes.

Finally, patients should not be encouraged to treat themselves or buy medications directly from pharmacies. They should be made aware that these medications have to be prescribed and monitored by qualified physicians or endocrinologists. There should be strict regulations on pharmacies not being able to sell medications for hypogonadism without a prescription.

## 5. Conclusions

There is a need for consensus on diagnostic and treatment guidelines for MH in India, including recommendations for regulating TRT dosage. It is recommended that for patients with functional hypogonadism or low levels of testosterone, TRT be initially administered for a short duration as a therapeutic trial. After reevaluation, if the levels are normal, therapy can be discontinued. For patients aged >65 years, TRT should be administered in the short-term. Additionally, patients should be made more comfortable to be able to discuss their symptoms.

Because a definitive diagnosis is impossible until an endocrinologist referral, it is also important that sufficient priority is given by physicians when diagnosing hypogonadism and in avoiding underdiagnosis. These needs can be addressed by devising continuing medical education programs to create awareness about MH among physicians.

## Figures and Tables

**Table 1:** Expert opinion on culture specific factors related to male hypogonadism.

(1) Most men are generally shy about discussing their sexual problems and tend to schedule multiple appointments for medical consultation
(2) Personality differences also impact men's willingness to report sexual dysfunction; for example, some men are vocal, while others tend to consider sexual dysfunction normal and not requiring medical consultation. Rushing in outpatient departments also deters men from being vocal about sexual dysfunction
(3) Hypogonadism, especially in older adults, goes unreported because most patients consider sexual dysfunction normal and part of the aging process. (i) After the age of 50 years, most Indian men do not complain of the problems related to hypogonadism, that is, sexual dysfunction; therefore, the incidence of MH in India is not an accurate indicator of the true prevalence
(4) Although there is no noticeable geographic skewness, a panelist mentioned that educated patients from north India are more comfortable discussing their sexual health than patients from the rest of the country
(5) Most Indian physicians are not trained in the etiquette of taking a sexual history or in the subject of MH. This leads to suboptimal clinician suspicion and screening
(6) A window of opportunity exists in mid-life: men who visit doctors with symptoms of prostatism must be screened for MH

**Table 2:** Expert opinion on medical history assessment.

During medical history assessment, several factors may indicate presence of low testosterone levels, including
Sexual history
(i) Lack of nocturnal erection
(ii) Absence of premature ejaculation
(iii) Lack of libido
Medical history
(i) Comorbidities involving lung, heart, liver, and kidney disorders
(ii) History of gynecomastia
Psychological history
(i) Anxiety or depression, and use of drugs for such conditions
Medication history
(i) Medical history of hormonal medication (before hormonal screening)
(ii) Use of antihypertensive drugs, especially propranolol
(iii) Withdrawal of any testosterone therapy
(iv) Use of dopaminergic drugs
Surgical history

**Table 3:** Diagnostic algorithm for late-onset hypogonadism.

Symptoms of hypogonadism (physical examination, history, and score)	Measure total testosterone	Low (<231 ng/dL)	Elevated LH and FSH	Exclude contraindications for testosterone	(i) Trial of testosterone; (ii) Monitor response
				Exclude pituitary and other causes	
		Borderline (231–346 ng/dL)	Calculate free testosterone	Low (<52 pg/mL)	Check for elevated LH and FSH
				Normal	Seek other causes
		Normal (>346 ng/dL)	Seek other causes of the symptoms		

Adapted from Kim et al., 2011 [19]. LH, luteinizing hormone; FSH, follicle-stimulating hormone.

**Table 4:** Indications for free testosterone testing in LOH.

Guideline	Threshold TT
EAU, EAA, ASA, ISSAM	230 ng/dL
AUA	300 ng/dL
AACE	200 ng/dL

AACE, American Association of Clinical Endocrinology; ASA, American Society of Andrology; AUA, American Urological Association; EAA, European Academy of Andrology; EAU, European Association of Urology; ISSAM, International Society for the Study of Aging Male; LOH, late-onset hypogonadism; TT, total testosterone.

**Table 5:** Expert opinion on testosterone replacement therapy.

(1) Oral preparations and gels are used less frequently because of lack of awareness, limited availability, and cost
(2) Long-acting injectables are frequently used but are difficult to administer
(3) Clinicians should be aware of rare side effects such as microembolism, pulmonary embolism, and anaphylaxis
(4) The potential impact of testosterone on spermatogenesis should be considered while planning therapy
(5) The use of a short-acting aqueous preparation, an injectable, or an oral preparation should be preferred as the initial dose of therapy
(6) Patients can later be shifted to long-acting preparations if they desire, once safety and tolerability have been demonstrated with shorter-acting testosterone
(7) Clinicians should discuss all available options with the patient and choose the preferred option through a process of informed and shared decision-making

**Table 6:** Testosterone preparations.

Formulation	Dosage	Points to consider
Injectable agents		
For quicker benefits		
Aqueous suspension of testosterone	25–50 mg every 1–2 weeks	Increased frequency of administration
Medium duration		
Testosterone enanthate	250 mg every 2–3 weeks	Wide fluctuation in testosterone levels
Testosterone cypionate	200 mg every 2–3 weeks	Multiple injections
Testosterone propionate	100 mg every 2 weeks	Relatively higher risk of polycythemia
Longer duration		
Testosterone undecanoate	1000 mg every 10–14 weeks^*∗*^	Pain at the injection site Risk of venous thrombosis

Oral agents		
Testosterone undecanoate	40–80 mg BID/TID with meals	Variable absorption; multiple doses
Oral testosterone undecanoate is a 17-*β*-undecylate molecule that is not hepatotoxic, and the variability in absorption with fatty meals is negligible		

Buccal agents		
Buccal bioadhesive testosterone tablets	30 mg controlled-release bioadhesive tablets BID	Gum-related adverse events in 16% of treated men

Topical agents		
Testosterone gel	Available in sachets, tubes, and pumps	Possible transfer during intimate contact Daily administration

Transdermal agents		
Transdermal testosterone patch	1–2 patches, designed to normally deliver 5–10 mg testosterone over 24 hours, applied every day on nonpressure areas	Skin irritation at the application site

Subcutaneous		
Surgical implants	2–6 pellets implanted subcutaneously	Require surgical incision for insertion Pellets may extrude spontaneously

BID, twice daily; TID; thrice daily.

## Data Availability

Not applicable as this is a review article.

## Abbreviations

BSSM: British Society for Sexual Medicine
EAU: European Association of Urology
ED: Erectile dysfunction
FSH: Follicle-stimulating hormone
IIEF: International Index of Erectile Function
ISSM: International Society for Sexual Medicine
LH: Luteinizing hormone
LOH: Late-onset hypogonadism
MH: Male hypogonadism
SHBG: Sex hormone-binding globulin
T2DM: Type 2 diabetes mellitus
TRT: Testosterone replacement therapy
TU: Testosterone undecanoate.
